# Cone photoreceptor dysfunction in retinitis pigmentosa revealed by optoretinography

**DOI:** 10.1073/pnas.2107444118

**Published:** 2021-11-18

**Authors:** Ayoub Lassoued, Furu Zhang, Kazuhiro Kurokawa, Yan Liu, Marcel T. Bernucci, James A. Crowell, Donald T. Miller

**Affiliations:** ^a^School of Optometry, Indiana University, Bloomington, IN 47405

**Keywords:** retinitis pigmentosa, optoretinography, photoreceptors, adaptive optics, optical coherence tomography

## Abstract

Retinitis pigmentosa (RP) is the most common group of inherited retinal degenerative diseases, whose most debilitating phase is cone photoreceptor death. Perimetric and electroretinographic methods are the gold standards for diagnosing and monitoring RP and assessing cone function. However, these methods lack the spatial resolution and sensitivity to assess disease progression at the level of individual photoreceptor cells, where the disease originates and whose degradation causes vision loss. High-resolution retinal imaging methods permit visualization of human cone cells in vivo but have only recently achieved sufficient sensitivity to observe their function as manifested in the cone optoretinogram. By imaging with phase-sensitive adaptive optics optical coherence tomography, we identify a biomarker in the cone optoretinogram that characterizes individual cone dysfunction by stimulating cone cells with flashes of light and measuring nanometer-scale changes in their outer segments. We find that cone optoretinographic responses decrease with increasing RP severity and that even in areas where cone density appears normal, cones can respond differently than those in controls. Unexpectedly, in the most severely diseased patches examined, we find isolated cones that respond normally. Short-wavelength–sensitive cones are found to be more vulnerable to RP than medium- and long-wavelength–sensitive cones. We find that decreases in cone response and cone outer-segment length arise earlier in RP than changes in cone density but that decreases in response and length are not necessarily correlated within single cones.

## Significance

Many blinding diseases afflict photoreceptors, specialized cells in the retina that capture and transduce light to initiate vision. Biomarkers that are sensitive to photoreceptor health are crucial for early detection and effective treatment monitoring of these diseases yet remain elusive. Here, we develop an optical biomarker, based on optoretinographic photoreceptor responses to light stimulation, that reflects the degree of dysfunction of individual cone photoreceptors in patients with retinitis pigmentosa (RP), the most common inherited retinal degenerative disease. Our results show that this biomarker may be beneficial for assessing the functionality of remaining retinal cells in RP patients and for assessing efficacy of treatments such as gene therapy and stem cell transplantation for RP and other diseases afflicting photoreceptors.

Retinitis pigmentosa (RP) is the most common group of inherited retinal degenerative diseases that lead to irreversible vision loss (1–3). It is characterized by progressive loss of peripheral vision that is separated from a shrinking island of healthy central vision by a transition zone (TZ) of reduced vision. As the disease progresses, the healthy island contracts, and the TZ migrates inwards. Degeneration typically starts in rod photoreceptors with early manifestation of nyctalopia and visual field loss then progresses to cone photoreceptors (4–6). Gene mutations of RP occur primarily in rods, resulting in extensive rod loss, but the loss of cones has a far more debilitating effect on vision. Cones mediate photopic vision and are essential for high visual acuity and color discrimination.

Recent advancements in gene replacement therapy, neuroprotective strategies, and stem cell therapy have heralded a new era for saving photoreceptors—cones in particular—in patients with RP and other inherited retinal degenerative diseases (7–9). All of these treatments as well as the disease itself act at the cellular level, yet methods for use in human subjects to assess the functional consequences of these changes on photoreceptors at this microscopic scale are limited.

Cone function (the ability to capture and transduce photons into visual signals) is routinely examined using perimetry and electroretinography (ERG) (1, 10). These methods are effective at indicating how the disease affects cone function, but they measure responses of aggregations of cones. Perimetric sensitivity is typically mediated by the tens to hundreds of cones that are affected by the stimulus and the many downstream neurons, so decreases may not even be caused by receptoral damage. Microperimetry has recently been enhanced with adaptive optics (AO) for improved resolution and with high-speed active tracking for correcting eye motion; these enhancements permit linking the visual percept associated with the activity of single cone cells (11), but this method (like all perimetry) is inherently subjective, requiring feedback from the subject, and sequential, stimulating one cone at a time. ERG and multifocal ERG are insensitive to the local spatial variations in disease progression that occur in RP because the measured electrical signal is generated by the combined responses of thousands to millions of the cone photoreceptors in the retina. In contrast, structural measurements made by clinical optical coherence tomography (OCT) and autofluorescence imaging enable considerably better spatial resolution and reproducibility, but they do not directly assess function (8).

Recent technological advances in high-resolution retinal imaging—AO in particular—have enabled cellular-level studies of RP in the living human retina. Studies using AO flood illumination and AO scanning laser ophthalmoscopy have shown cellular structural changes (12–19), including during treatment to slow RP-related retinal degeneration (20). These structural measurements have also been combined with microperimetric functional measurements (21). However, functional changes in individual cones have yet to be studied in RP (or indeed in any other cause of irreversible vision loss [e.g., age-related macular degeneration, diabetic retinopathy, or progressive myopia]).

**Fig. 1.**
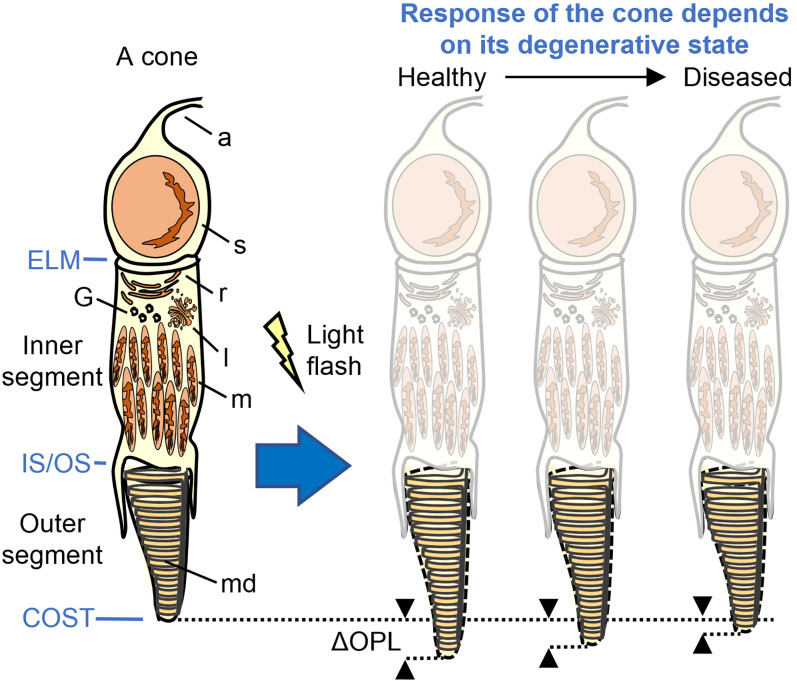
It is known that cone OSs briefly increase their optical path length (e.g., elongate as shown) when exposed to a light flash. We hypothesize that this response decreases with severity of the disease, with cones responding less when degenerating than when healthy. a, axon; ELM, external limiting membrane; G, Golgi; l, lysosome; m, mitochondria; md, membranous discs; r, reticulum; and s, soma.

To address this need, we investigate an optical biomarker to quantify the dysfunction of individual cone cells in RP subjects. We combine AO and phase-sensitive OCT (PS-AO-OCT) (22–26) to measure nanometer-scale optical path length changes (ΔOPL) occurring inside photoreceptors during photoactivation (photoreceptor response to light; Fig. 1). Unlike the previous AO imaging methods applied to RP, our PS-AO-OCT method has sufficient resolution to reveal the three-dimensional reflectance profile of individual cones (structure) and sufficient sensitivity to detect photostimulated OPL changes as small as 5 nm in the same individual cones (function).

The ΔOPL cone response that we measure is part of the cone optoretinogram, the optical analog of the electroretinogram but based on changes in optical properties of cones instead of on their electrical activity when stimulated with light (27). Prior work by us and others has established the cone optoretinogram’s ΔOPL response as a correlate of cone function in healthy subjects (23–26, 28–34). This includes ΔOPL measurements with PS-AO-OCT to classify individual cones into the three spectral types that are sensitive to short- (S), medium- (M), and long- (L) wavelength light (24), to quantify the spectral sensitivity of S, M, and L cones (31), to reveal how color vision phenotype and genotype manifest in individual cones cells (32), to elucidate steps of the phototransduction cascade (26), and to reveal functional responses of both rods and cones (30). Intensity manifestations of ΔOPL responses as measured with AO flood illumination fundus cameras and AO scanning laser ophthalmoscopes have been used as optical measures of cone function (28, 33, 34) and photopic luminosity (29). Collectively, these studies provide strong support for the use of the cone optoretinogram’s ΔOPL response as a measure of cone function.

**Fig. 2.**
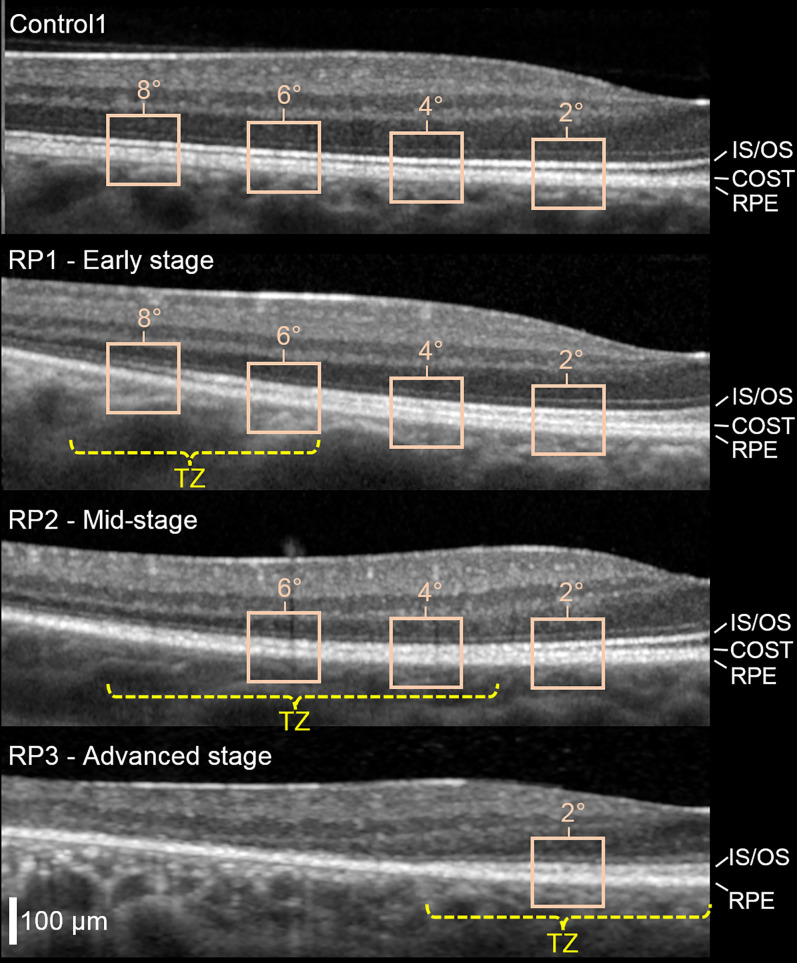
Effect of RP disease on cone photoreceptor hyperreflective bands as observed in clinical OCT images. Cross-sectional (B-scan) images obtained with Spectralis OCT reveal the TZ between healthy and severely diseased retina. Shown are one of the controls and the three RP subjects with different stages of RP disease. Foveal center is at the right edge of image; images subtend 10° along the temporal horizontal meridian. The TZ (yellow) is defined as the area between the two retinal eccentricities where the COST and IS/OS hyperreflective bands merge with the underlying RPE band and thus become indistinguishable in the B-scan (12). Superimposed on the OCT images are the retinal locations (beige boxes) we imaged with AO-OCT (2, 4, 6, and 8°). RPE, retinal pigment epithelium.

Here, we improve and extend this work to measurement of abnormal or impaired function (dysfunction). We quantify responses and classify cells in different retinal locations and in different subjects with varying degrees of disease severity. We describe disease severity of a given retinal location in a particular eye by its location with respect to the TZ in that eye, with severity increasing from the inner to the outer edge of the TZ. We define TZ operationally (following ref. 12) as the region of retina between two eccentricities: one at which the cone photoreceptor outer segment (OS, the cell compartment packed with photosensitive pigment to capture light) begins to abnormally shorten and another at which it disappears. These are estimated from a clinical OCT image as the terminations of the hyper-reflective bands corresponding to the cone outer segment tip [COST, also referred to as the interdigitation zone (35)] and photoreceptor inner-segment/outer-segment junction [IS/OS, also referred to as the ellipsoid zone (35)] (see Fig. 2).

We test the hypothesis that the cone ΔOPL response to light decreases with increasing disease severity (see Fig. 1; [i.e., that the response of a given cone cell is influenced by its location with respect to the TZ in that eye]). More specifically, based on cone OS length derived from clinical OCT images we hypothesize that cone responses should be normal in the healthy central island (if present) and gradually decrease across the TZ to zero at its outer boundary, where cells show no OS. To test at the single-cone level, we compare the individual ΔOPL response and OS length of cones in the same retinal patch. We measured cone ΔOPL responses at 1) the same retinal location in different RP subjects with varying disease severity (early to advanced, based on proximity of the TZ to the fovea and clinical visual sensitivity) and 2) multiple retinal patches in the same RP subject but located differently with respect to the TZ. To establish confidence limits, we compare the results with those from healthy controls. Having established a relationship between ΔOPL and clinical measures of cone health, we examine whether particular cone spectral classes (S, M, and L) or individual cones within a class are more vulnerable in RP.

## Results

### Clinical and AO-OCT Measurements.

Clinical and AO-OCT measurements were obtained on six subjects: three RP subjects (RP1, RP2, and RP3; all autosomal recessive) and three age-matched controls (Control1, Control2, and Control3). The three controls fell in the normal population range (95% confidence interval [CI]) for cone density (measured with AO-OCT), cone OS length (measured with Spectralis OCT), visual sensitivity (measured with Octopus 900), and had normal color vision (measured with pseudoisochromatic plates and anomaloscope). Details of these measurements are in *SI Appendix*, Figs. S1–S3 and Table S1. The three controls also had ΔOPL responses that fell in the 95% CI of a separate, larger control group of responses from 14 healthy subjects with measurements at 3.7° temporal eccentricity (32) (*Materials and Methods*).

Clinical OCT images (Fig. 2) and perimetry maps (*SI Appendix*, Fig. S3) of the three RP subjects reveal the characteristic central healthy island in early-to-moderate stages of RP. These subjects were rank ordered by disease severity and labeled RP1 (early stage), RP2 (midstage), and RP3 (advanced) based on the proximity of the TZ to the fovea and reduction in visual sensitivity. B-scans from clinical OCT images reveal the location of the TZ (RP1: 5.5 to 9°; RP2: 3 to 8.5°; and RP3: 0 to 4° retinal eccentricities) and its position relative to the 2, 4, 6, and 8° retinal eccentricities imaged with AO-OCT along the temporal horizontal meridian as shown in Fig. 2. For RP3, the TZ covers the fovea. Color vision was normal in subjects RP1 and RP2, but RP3 had a tritan defect on the anomaloscope test. *SI Appendix*, Table S1 summarizes our clinical measures.

During AO-OCT imaging, we used three stimuli at different wavelengths across the visible spectrum (450, 528, and 637 nm). These allowed us to test the functional response of all cone types to better separate the responses of diseased cones and to avoid ambiguity between S, M, and L cones and possible nonabsorbing or nonresponding cones.

**Fig. 3.**
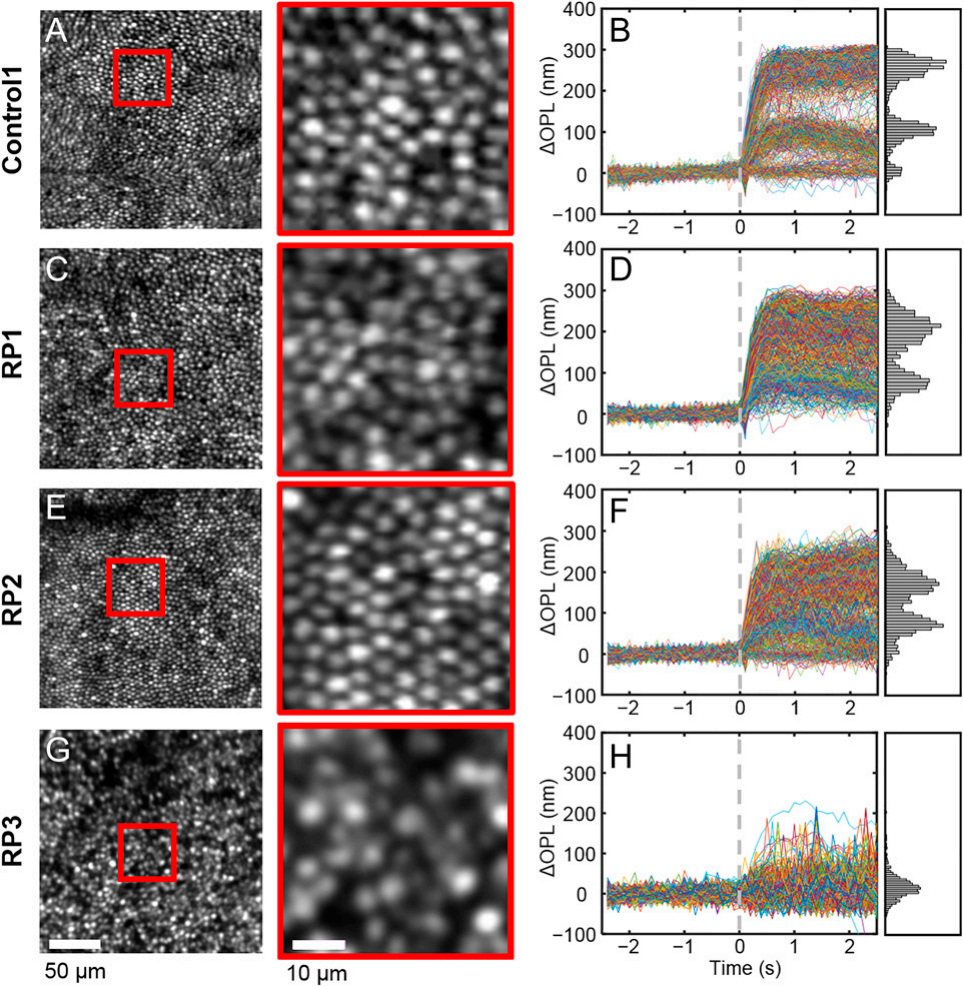
Cone photoreceptor structure and function. AO-OCT en face images of the cone mosaic at 2° eccentricity in one of the controls (*A*, and* B*) and the three RP subjects: RP1 (*C* and *D*), RP2 (*E* and *F*), and RP3 (*G* and *H*). Magnified views are shown for cones in red boxes. Cone densities are 31,491 (Control1), 22,750 (RP1), 25,258 (RP2), and 14,041 (RP3) cones/mm^2^. Traces of individual cones’ ΔOPL responses to 637-nm stimulus are shown in *B*, *D*, *F*, and *H*. Traces are randomly color coded, and histograms of peak responses (average ΔOPL from 0.75 to 1.25 s after stimulus) are shown to the right of each plot.

Fig. 3 presents our structural (*Left Column*) and functional (*Right Column*) measurements of cone cells acquired with AO-OCT at 2° retinal eccentricity in the three RP subjects and one control (Control1). For brevity, photostimulation data are shown for only one of the three stimuli. The 2° location fell into the central healthy area of RP1 and RP2 and inside the TZ of RP3. As evident in Fig. 3 *A*, *C*, and *E*, the en face intensity images of subjects RP1 and RP2 show regular cone mosaics, similar to that of Control1. In order to substantiate this observation, we measured the cone density and aggregate OS length in RP1 and RP2 and compared them to the normal population (*SI Appendix*, Figs. S1 and S2).

At 2°, RP1 and RP2 indeed have cone densities within the normal range and OS lengths that align to the lower edge of the 95% CI, similar to that of Control1. Interestingly, however, functional measures of the 2° cones, as captured by their ΔOPL traces after photostimulation, reveal notable differences (Fig. 3 *B*, *D*, and *F*) between control and RP subjects. The control subject exhibits three distinct peaks in cross-section [indicating three cone spectral sensitivity clusters, as expected in normals (24, 32) and described in *Principal Component Analysis*], whereas RP1 and RP2 each show just two reduced peaks. For the late-stage RP subject (RP3) (Fig. 3 *G* and *H*), cone density and OS length fall below the normal population ranges (*SI Appendix*, Figs. S1 and S2), and functional measures of the remaining cones have a single, reduced peak, suggesting that almost all of them are severely compromised.

### Principal Component Analysis.

**Fig. 4.**
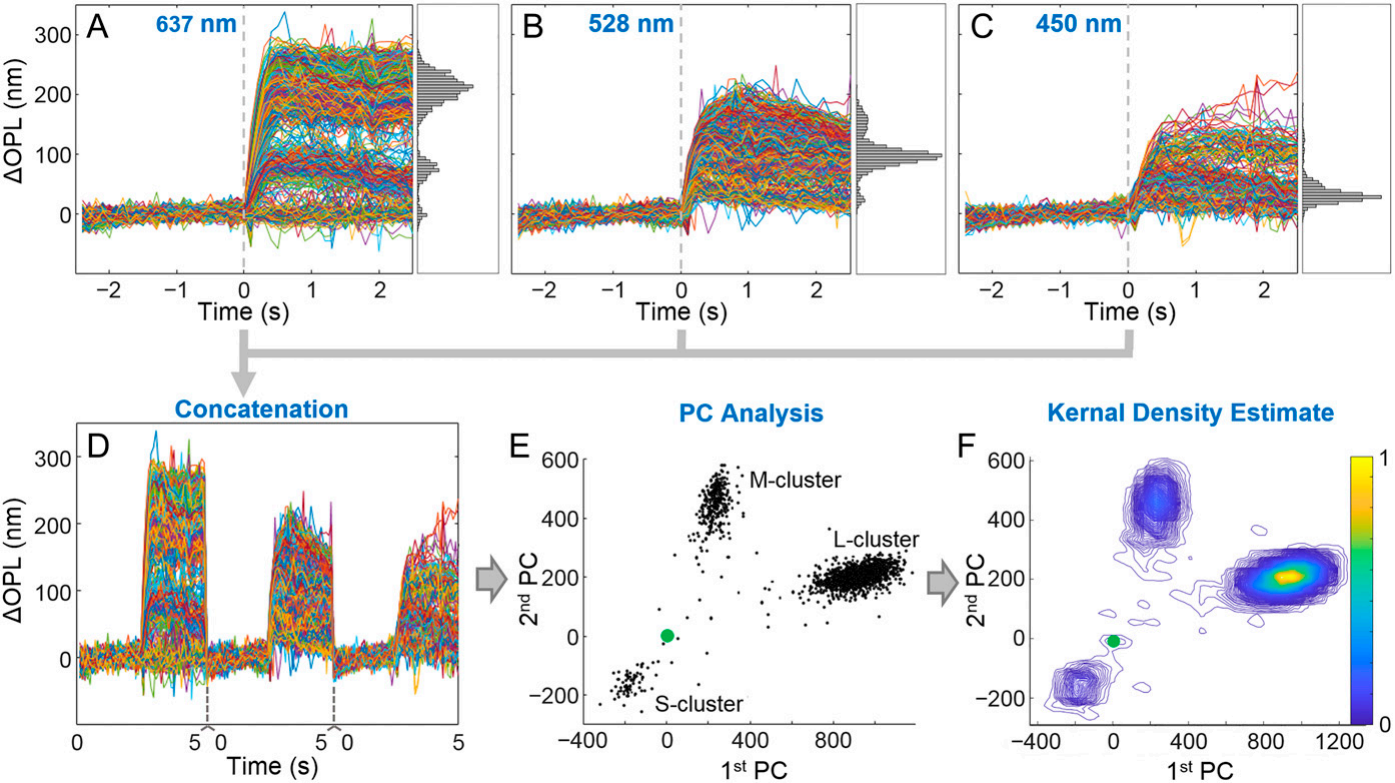
Key steps for processing the cone response traces, illustrated using control subject (Control3). The ∼1,000 cones respond differently depending on the wavelength of the flash stimulus: 637 (*A*), 528 (*B*), and 450 nm (*C*). Flash stimulus occurs at time equal to 0 s. Individual cone traces are randomly colored. The 5-s-long cone traces in *A*, *B*, and *C* are concatenated to form 15-s long traces (*D*) that are then transformed to PC space to reveal cone clusters (*E*), one for each of the three cone spectral types (S, M, and L). (*F*) A kernel density estimate is applied to the PC plot in *E* to locate the peak of each cluster, whose relative position to the origin [0,0] (green marker) is a measure of each spectral type’s collective response.

To facilitate analysis of the cone responses and to classify cones by spectral type, we remapped the combined ΔOPL traces from the three stimuli (Fig. 4 *A*–*D*) using principal component (PC) analysis (see details in *SI Appendix*, *SI Materials and Methods*). We then plotted the values of the first and second PC components, which captured most of the variance in the cone responses (Fig. 4*E*). We identified the S-, M- and L-cone clusters following our previous work (24). Finally, we estimated the center of each cluster by smoothing the two-dimensional (2D) distribution of cone responses in the PC space (approximately 1,000 cones per location) with a kernel density estimate (Fig. 4*F*) and determining the peak density location of the smoothed distribution for each cluster. In the PC plots, larger (radial) distances from the origin correspond to stronger cone responses, and the origin [0,0] is the location of nonresponding cones. M and L cones generally respond more strongly than S cones to the three stimuli used. Angular directions from the origin are determined by spectral sensitivities of the cones, thus S, M, and L cones have different angular positions.

### Early-Stage RP Subject.

**Fig. 5.**
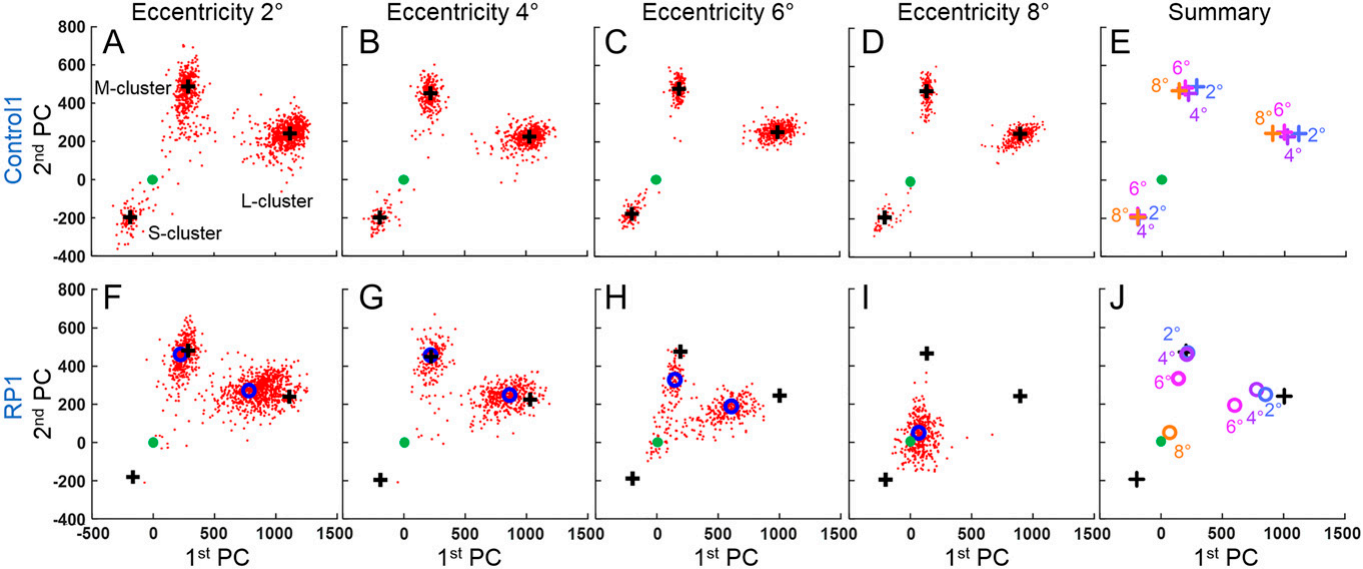
Cone ΔOPL response decreases as disease severity increases with retinal eccentricity in RP1. The cone response to the three LED stimuli is projected along the first two principal components following the process shown in Fig. 4. The first row is the age-matched control at 2 (*A*), 4 (*B*), 6 (*C*), and 8° (*D*) retinal eccentricity. (*E*) All cluster peaks from Control1 overlaid in one plot. Each color corresponds to one location. The second row is RP1 at 2 (*F*), 4 (*G*), 6 (*H*), and 8° (*I*) retinal eccentricity and a summary plot of the four previous locations (*J*). In all plots, solid green marker indicates [0,0] location. Black plus markers are the cluster positions of the control (Control1), as computed from the kernel density estimate. Open blue markers are cluster positions of RP1, also computed from the kernel density estimate. In *J*, black plus markers are the average cluster positions of S, M, and L cones of Control1 over the four retinal locations in *A*–*D*.

Fig. 5 presents cone responses in PC space from RP1 and their age-matched control. For the control, we see three distinct response clusters corresponding to the three cone spectral types (S, M, and L). The narrow radial extents of the clusters relative to their distances from the origin [0,0] indicate that almost all cones responded to light. The clusters are present at all four retinal eccentricities (2, 4, 6, and 8°) and shift little between eccentricities, as indicated in Fig. 5*E* by their close proximity to each other and location within the 95% CI of the 14-subject control group (specified in next paragraph).

**Fig. 6.**
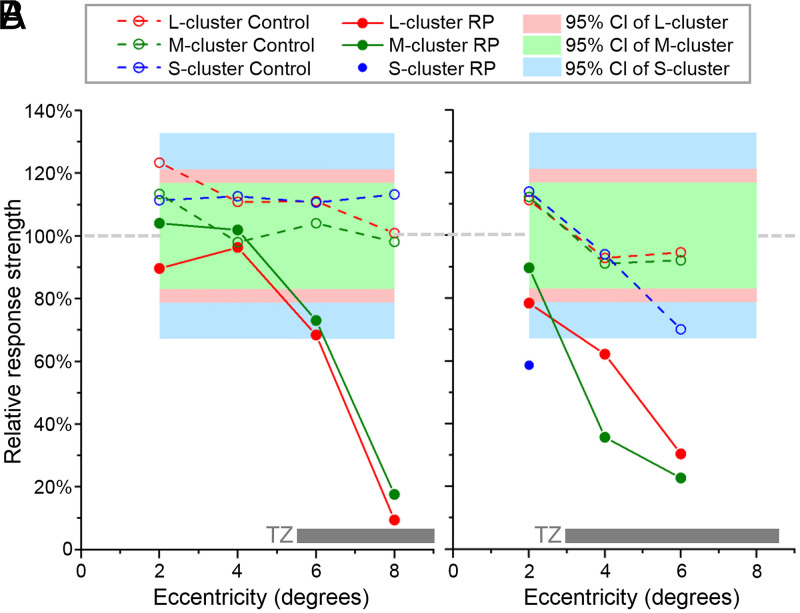
Relative ΔOPL response strength of cones as a function of retinal eccentricity and cone spectral type for RP1 (*A*) and RP2 (*B*) subjects. Response strength is relative to the 14-subject control group (100%). The 95% CIs of S, M, and L clusters are for the 14-subject control group and are centered on 100%. Traces are color coded by spectral type: S (blue), M (green), and L (red). For reference, the gray horizontal bars at the bottom denote the location and range of the TZs of the RP subjects.

Conversely, RP1 exhibits only two distinct groups, corresponding to controls’ M and L clusters; this subject appears to have no functioning S cones. These clusters are broader and shifted toward the origin [0,0], indicating that RP1 cone responses are generally more variable and weaker. To facilitate comparison between cone spectral types and subjects, we define the relative response strength as the ratio of the distance of a cluster center from the origin to that of the average of the corresponding spectral-type clusters across the 14-subject control group; a relative response strength of 100% thus represents a normal response, whereas 0% represents no response to light. Using this relative response strength, the 95% CIs of the 14-subject control group are 67 to 133% (S), 83 to 117% (M), and 79 to 121% (L). At 2 and 4° (Fig. 5 *F* and *G*), the relative response strengths of the L and M clusters in RP1 are 96 and 90% (L cones) and 102 and 104% (M cones), values that are well within the controls’ 95% CI. At 6°, the corresponding values are 68 and 73%, both falling below the 95% CI of the 14-subject control group (Fig. 5*H*). At 8° (Fig. 5*I*), RP1 has only one cone cluster, so we are unable to distinguish the cone spectral types. The relative response strengths of the cone clusters are summarized in Fig. 6*A*, plotted as a function of retinal eccentricity and cone spectral type (where possible).

**Fig. 7.**
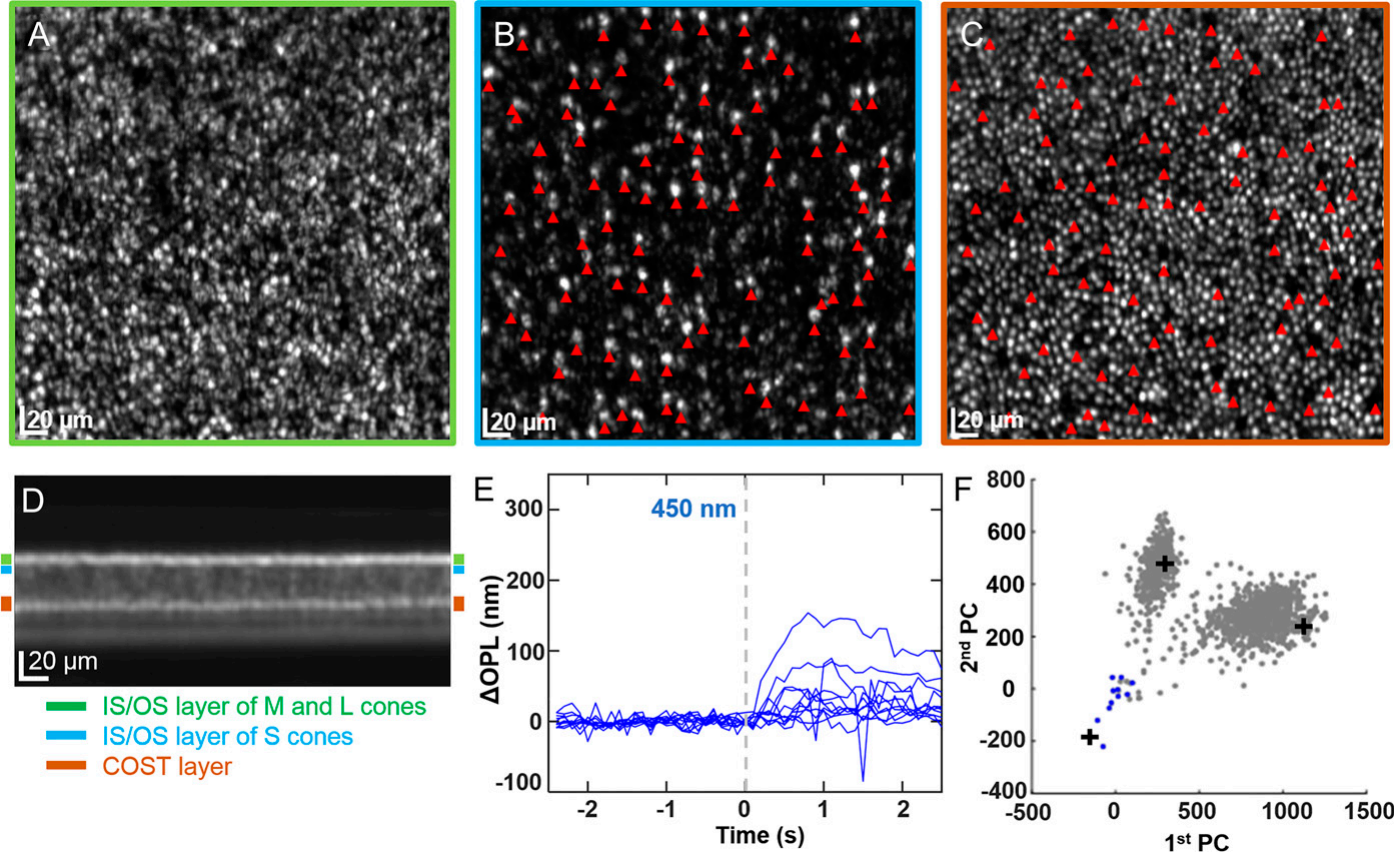
Structural and functional evidence of S cones at 2° eccentricity in subject RP1. En face images are projections over different depths in the photoreceptor layer of the AO-OCT volume: IS/OS band (*A*), immediately below the IS/OS band (*B*), and COST band (*C*). Bands were determined from the projected cross-sectional image of the volume as labeled in *D*. The three colored rectangles specify the projection ranges and have vertical widths of 7 (green), 5 (blue), and 10 μm (brown). In *B*, the relatively sparse array of bright punctuated reflections (many indicated by red arrowheads) are consistent with S cones, whose inner segments are known to be 10% longer than M and L cones (36). For this retinal patch, the apparent S-cone IS/OS reflection is shifted about 5 μm deeper than that of M and L cones. The pattern of red arrowheads in *B* are superimposed in *C*, revealing that most S cones lack a reflection in the COST band. (*E*) ΔOPL response of the 11 strongest responding S cones to the 450-nm stimulus are shown, selected from the 45 S cones that were manually identified based on a bright reflection in *B* and a COST reflection at a depth shallower than the COST band in *C* (see details in *Early-Stage RP Subject*). (*F*) In PC space, the 11 apparent S-cones are colored blue, and those previously identified in Fig. 5*F* are colored gray. For reference, the black plus markers are the cluster positions of the control (Control1) at 2° eccentricity as computed from the kernel density estimate.

RP1’s foveal color vision was normal, so the apparent absence of functional S cones at 2° (Fig. 5*F*) is of particular interest. To investigate this apparent discrepancy, we took advantage of the fact that S cones can be anatomically distinguished by their slightly longer inner segments (36) and that consequently their IS/OS reflections are shifted slightly deeper in the retina compared to those of M and L cones (37). Fig. 7 illustrates our process. Fig. 7*A* contains the projected image of the cone mosaic obtained from segmentation of the AO-OCT volume at the depth of the IS/OS junction of M and L cones. We repeated the segmentation at a slightly greater depth (∼5 μm), where we expected to find the IS/OS junction of S cones if they were present. This is shown in Fig. 7*B* and reveals a sparse array of bright reflections; we take these to indicate the presence of S cones, and the location of 94 of them are marked by red arrowheads. In Fig. 7*C*, we show the segmentation of the same volume at the depth of COST. While M and L cones still have reflections in the COST image, the S cones just identified generally do not (indicated by the dark gaps at their locations in the COST image). This lack of COST reflections suggests that the S-cone tips are either lost or displaced. We manually inspected each S cone in the volume and found that only 49 (of the 94 identified) had a COST reflection, and all but 1 of those occurred at a shallower depth than those of M and L cones; the OSs of these 49 S cones were on average 9 ± 2 μm shorter than those of M and L cones. Having located their OSs, we were able to perform the PC analysis on these 49 S cones. The 11 strongest responding cones of the 49 are plotted in Fig. 7 *E* and *F*; the remaining responses were not significantly different from zero (<±10 nm, that is less than ±2 SD of the sensitivity of our measurement). As evident in the PC plot, even these 11 strongest cones (colored blue) cluster about the origin [0,0], with only two cones approaching the average S-cone response of the 14-subject control group (plus symbol in plot). To summarize, we find evidence of S cones at 2° in RP1, but most have abnormally shortened OSs and little or no response to light.

### Midstage RP Subject.

**Fig. 8.**
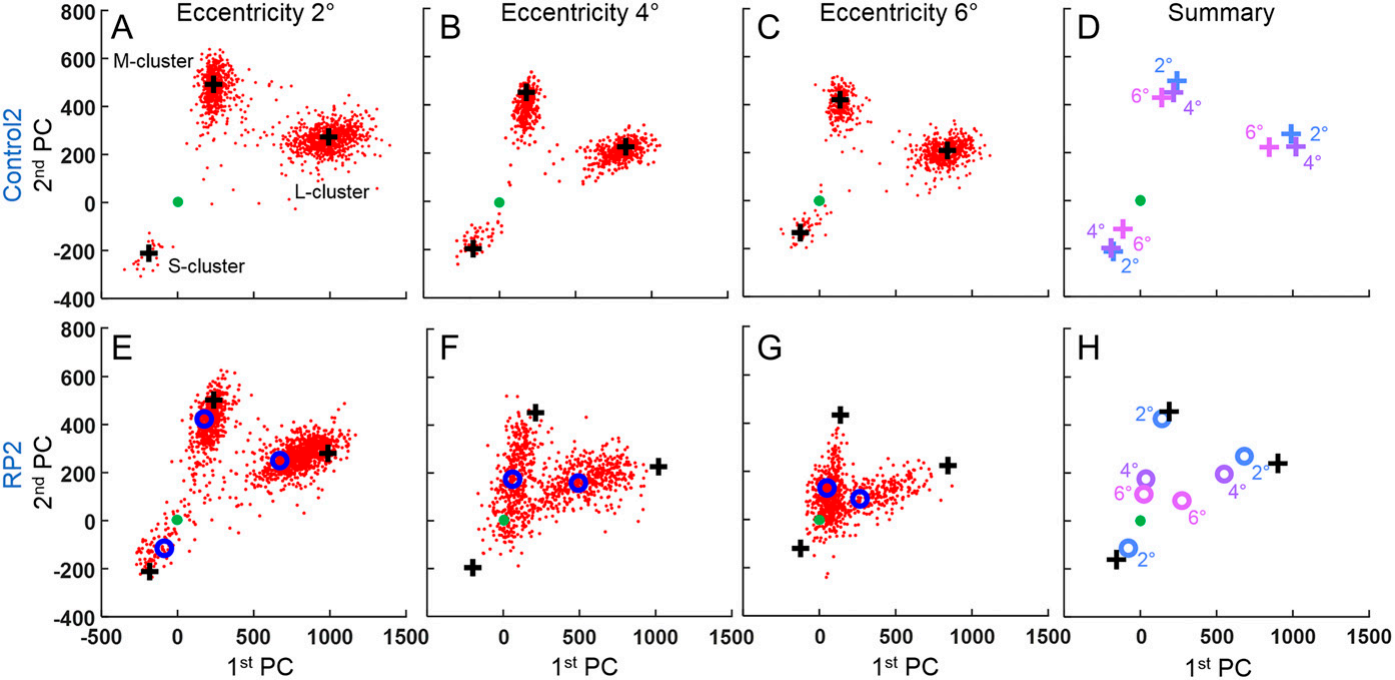
Cone ΔOPL response decreases as disease severity increases with retinal eccentricity in RP2. The cone response to the three LED stimuli is projected along the first two principal components following the process shown in Fig. 4. The first row is the age-matched control at 2 (*A*), 4 (*B*), and 6° (*C*) retinal eccentricity. (*D*) All cluster peaks from Control2 overlaid in one plot. Each color corresponds to one location. The second row is RP2 at 2 (*E*), 4 (*F*), and 6° (*G*) retinal eccentricity and a summary plot of the three previous locations (*H*). In all plots, solid green marker indicates [0,0] location. Black plus markers are the cluster positions of the control (Control2) as computed from the kernel density estimate. Open blue markers are cluster positions of RP2, also computed from the kernel density estimate. In *H*, black plus markers are the average cluster positions for S, M, and L cones of Control2 over the three retinal locations in *A*, *B*, and *C*.

Our cone response results in PC space for RP2 and their age-matched control are shown in Fig. 8. The corresponding relative response strengths in RP2 are summarized in Fig. 6*B*, plotted as a function of retinal eccentricity and cone spectral type. As shown in Fig. 8*D*, clusters’ peak positions in the control subject are relatively stable with retinal eccentricity and locate within the 95% CI of the 14-subject control group.

Results for RP2 are similar to those for RP1, except that RP2 does have an S-cone cluster in the PC plot at 2°. RP2’s clusters are increasingly shifted toward the origin [0,0]—indicating weaker responses to light—as eccentricity increases. At 2° (Fig. 8*E*), the relative response strength of the M cluster is 90%, falling within the controls’ 95% CI, while those of the L- and S-cone clusters are 78 and 59%, respectively, falling below the 95% CI. At 4° (Fig. 8*F*), relative response strengths for the L- and M-cone clusters are 62 and 36%, and at 6°, they are 30 and 23% (Fig. 8*G*), respectively. There was no evidence of S cones at 4° and 6°. L- and S-cone responses at 2° and all responses at 4° and 6° fall below the 95% CI of the control group. No measurements were made at 8° as a weak cone response was reached at 6°.

### Late-Stage RP Subject.

**Fig. 9.**
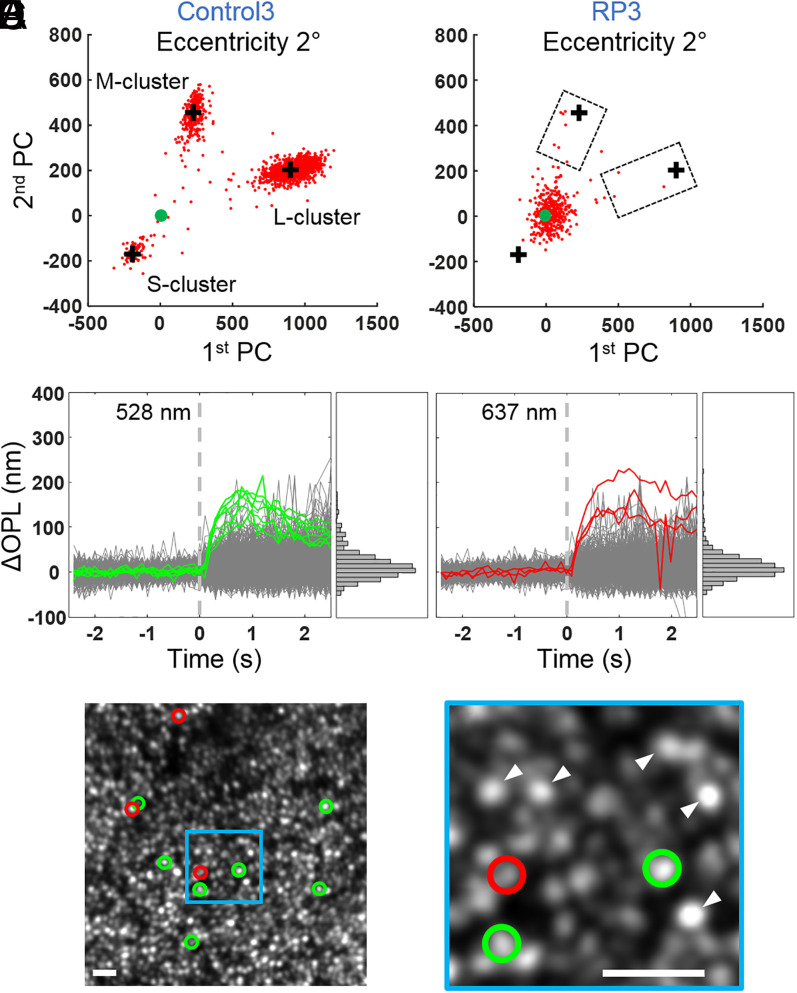
Cones in RP3 are severely compromised except for a small fraction that respond normally. ΔOPL response in PC space in age-matched control (Control3) (*A*) and RP3 at 2° retinal eccentricity (*B*). The cone response to the three LED stimuli is projected along the first two PC components. Solid green marker indicates [0,0] location. Black plus markers are the cluster positions of the control (Control3) as computed from the kernel density estimate. While most cones are clustered around [0,0] in *B*, 10 cones identified as M (green) and L (red) based on their PC mapping exhibit a strong response (inside the two dashed boxes), some sufficiently so that they are indistinguishable from cones in the control. The sides of the boxes closest to the origin [0,0] are located halfway (50%) between the origin and black plus markers of the control. Traces are shown of all cones’ ΔOPL responses to the 528- (*C*) and 637-nm stimuli (*D*); histograms of peak responses (average of ΔOPL from 0.75 to 1.25 s after stimulus) are shown to the right of each plot. In *C*, the strong response of the seven M cones (green) is distinct from the other cones’ responses (gray), confirming the PC analysis results. Similarly, in *D*, the strong response of the three L cones (red) is distinct from the other cones’ responses (gray). (*E*) These 10 cones were then mapped back to the AO-OCT en face image and labeled with green (M cones) and red (L cones) circles. (*F*) Close-up of a smaller area highlights three of the strongly responsive cones, two identified as M cones and one as an L cone. White arrowheads point to bright cones that did not respond strongly to the stimuli. (Scale bars in *E* and *F*, 10 μm.)

Fig. 9 presents cone responses from RP3 and their age-matched control. Due to the severity of the disease, cones respond substantially less in RP3 than in control subjects, even close to the fovea at 2°. We observe only one cluster, near the origin [0,0] in PC space, so we cannot categorize most cones by spectral type. A very small proportion of cone responses (10 out of more than 850 cones still present, 1.2%) were more than 50% of the distance from the origin to the cluster positions of the control (Fig. 9*B*). We classified these strongly responding cones by comparing their angular positions in the PC plot to the averages of the 14-subject control cluster centers (see also red and green colored cones in Fig. 9*E*): seven were M cones and three were L cones. Of these, four M cones and one L cone fell within the 95% CI of the 14-subject control group, thus these five cones were statistically normal. In Fig. 9*C*, the traces of the response from the seven M cones to 528 nm are plotted in green, while traces of the remaining cones are plotted in gray. The seven M cones exhibit significantly stronger responses than all other cones. In Fig. 9*D*, cone responses to 637 nm are plotted. Similarly, the three L cones, identified in the PC space, exhibit stronger responses than others.

## Discussion

We successfully characterized the responsiveness of single cone cells in subjects afflicted with RP and compared them to those in normal healthy subjects. To achieve this, we stimulated cone cells with brief flashes of light at different wavelengths and measured their resulting nanometer-scale changes in optical path length using PS-AO-OCT. We used PC analysis to examine the optical path length changes, allowing us to quantify each cone’s response strength and to distinguish S-, M-, and L-cone responses in normal and diseased eyes. Listed by topic, we discuss key findings from the study.

### Cone Response with Severity of Disease.

We tested the hypothesis that the cone ΔOPL response decreases with increasing disease severity, that is, that the response of a given cone cell is influenced by its location with respect to the TZ in that eye. In all cases examined, we found this to be true. By comparing ΔOPL responses at different retinal locations in the same RP subject (see Figs. 5*J* and 8*H* PC plots and Fig. 6 summary plots for the early- and midstage RP subjects), we observed the following important trend: responses decline precipitately with retinal eccentricity, consistent with the expected degradation of the outer retina in the TZ (Fig. 2).

Between subjects, the overall cone response decreases with disease progression from early to advanced, as evidenced most clearly by the results summarized in Fig. 6. RP1 shows a plateau of near-normal M- and L-cone responses at 2 and 4° retinal eccentricity followed by a rapid decline at larger eccentricities. RP2 shows only a similar rapid decline starting immediately at 2°. RP3 shows virtually no cone responses, with a single cluster around the origin [0,0] at 2° (Fig. 9*B*), the least-diseased location we imaged in this subject.

We find these overall trends are less predictive of individual cone responses in diseased compared to normal retina. In the control subjects, cone cells of the same retinal patch and same spectral type responded more or less similarly to the same light flash (compact cluster in PC space). In the RP subjects, the responses were more variable, with some cones seemingly more affected by the disease than others. This is particularly evident in the TZ. For example, cones in RP1 at 6° (Fig. 5*H*) and RP2 at 4° (Fig. 8*F*) show responses ranging from normal healthy cone responses to no response at all. It appears that ΔOPL responses of some cones are much more vulnerable to the disease than others.

It is curious that in some severely diseased patches we found isolated cones with ΔOPL response magnitudes that were above 50% of normal, and a few of these were statistically normal [e.g., in RP3 at 2° eccentricity (Fig. 9), we found seven such M cones and three such L cones]. As shown in Fig. 9*E*, these 10 cones do not appear to form a local cluster. What is it that renders these particular cones apparently immune—at least temporarily—to the dystrophy about them? This resistance might hold clues to the underlying (and unknown) causes of cone death of which several hypotheses have been proposed for RP. These include 1) toxins from the degeneration of surrounding rods (38); 2) cytotoxic factors released by glial cell activation (39); 3) oxidative damage (40–42); 4) loss of rod-derived trophic support (5, 43, 44); and 5) starvation and nutritional imbalance, driven by the insulin/mammalian target of rapamycin pathway (6, 45). Extending our imaging method to the cells that surround cones, in particular rod photoreceptors, that often contain the RP gene mutation or microglial cells that respond by invading the outer retina (46), would add additional power to test these hypotheses.

### Vulnerability by Cone Spectral Type.

It is well known that subjects with RP often suffer from acquired color vision deficiencies (M–L-cone pathway, S-cone pathway, or both) due to cone photoreceptor damage (47). The S-cone pathway is more commonly and more severely damaged than the M–L-cone pathway (47–49). Thus, we sought to determine if our RP subjects’ S, M, and L cones were differentially vulnerable.

In subjects RP1 and RP2, S cones were observed only at the closest foveal location that we imaged (2° eccentricity). For subject RP1, S cones had OSs that were notably shorter than M and L cones (see Fig. 7*C*) and ΔOPL responses that were considerably reduced (see Fig. 7 *E* and *F*). For subject RP2, S cones had OSs of normal length but responses that were reduced (a relative response of 59%; Fig. 6). M- and L-cone responses in both RP subjects decreased similarly, showing little evidence that one of these two cone types was more vulnerable to RP than the other. For these two RP subjects, S cones were at a more advanced stage of the degeneration than M and L cones, pointing to more compromised color vision in the S-cone pathway than the M–L-cone pathway. Interestingly, despite these losses, both RP subjects had normal color vision as measured with the pseudoisochromatic plates and anomaloscope, presumably because a sufficient number of S cones remained functioning in the fovea.

For subject RP3, we found no evidence of S cones in our AO-OCT dataset. We measured no S-cone cluster and found no cones with a displaced IS/OS reflection. These findings are consistent with the tritan defect we measured in this subject using the pseudoisochromatic plates and anomaloscope. While we were unable to distinguish M and L cones in the single cluster of cones centered on the origin [0,0] in PC space, the weak response of these cones indicates both M and L cones were severely compromised by the disease.

In general, we find that S cones were more vulnerable than M and L cones to RP in our subjects; this trend is consistent with previous reports using psychophysics and ERG (47–49), but now we can measure this acquired color vision deficiency at the level of single-cone cells as it evolves across the retina.

### Comparison of Structure and Function from Imaging.

Cone density is the most commonly used structural metric of cone photoreceptors in high-resolution retinal imaging studies of RP and has been reported significantly lower in these subjects (20, 50–52). However, cone density is inherently variable between subjects. In our study, we measured cone density at all retinal locations imaged and in subjects RP1 and RP2 found cone densities were within the normal range as reported in the literature (detailed in *SI Appendix*, Fig. S1). Only in subject RP3, who had the most severely diseased retina, did cone density fall significantly below normal (outside 95% CI). In contrast, cone ΔOPL responses as quantified in PC space were reduced, dramatically so at the larger retinal eccentricities for M and L cones and at all locations for S cones. Even retinal locations, where the average cone response was near zero [0,0], retained normal cone density, see Figs. 5*I* (RP1) and 8*G* (RP2).

Furthermore, we were struck by how disconnected the appearance of a cone cell in an en face AO-OCT image could be from its response to light (e.g., compare the appearances of the 10 strongest responding M- and L-cone cells in Fig. 9 *E* and *F* [RP3] to those of their weakly responding neighbors). We found that many cones that appeared structurally normal (bright punctate reflection in the en face image as for example those marked by white arrowheads in Fig. 9*F*) had diminished responses, sometimes dramatically so.

We found cone OS length measured with clinical OCT to be a more sensitive structural measure of disease severity than cone density. OS length was significantly reduced (falling outside the 95% CI) for eccentricities ≥4° for RP1 and ≥2° for RP2 and RP3 (detailed in *SI Appendix*, Fig. S2). Interestingly, our aggregate ΔOPL responses of M and L cones were no more sensitive than clinical OCT OS length measurements. Specifically, we measured significant reductions in M- and L-cone responses at ≥6° for RP1, ≥4° for RP2, and ≥2° for RP3 (see Figs. 6 and 9*B*); *SI Appendix*, Fig. S2 demonstrates that RP subjects’ OS lengths fall below the normal 95% CI at similar eccentricities to these. Similar sensitivity of the two metrics is perhaps not surprising as the capacity of a cone photoreceptor to hold photopigment increases with OS length and in turn increases the potential for a stronger hyperpolarization signal and a stronger ΔOPL response ( we examine this hypothesis at the level of individual cones in *Correlation of Individual Cone ΔOPL Response with OS Length*). In contrast to M and L cones, the S-cone response was significantly reduced at all locations and in all subjects measured, even in the supposedly healthy island of the retina (closer to the fovea than the beginning of the TZ).

### Correlation of Individual Cone ΔOPL Response with OS Length.

**Fig. 10.**
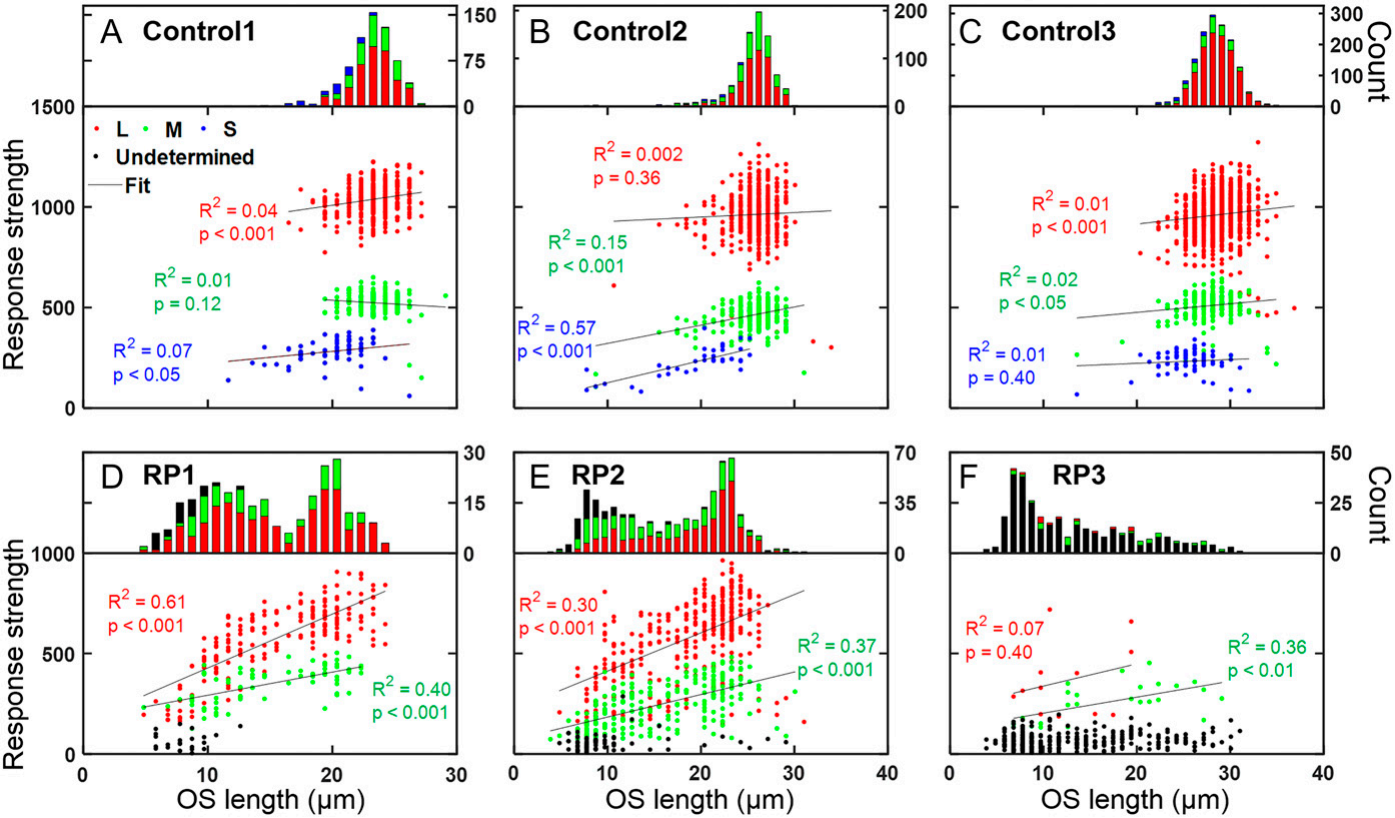
Cone OS length is a coarse predictor of cone ΔOPL response, a relationship that varies with severity of the RP disease. The magnitude of each cone’s response in PC space is plotted against OS length. Cones are classified as S (blue), M (green), L (red), and undetermined (black). The distribution of cone OS lengths is shown at the top of each plot and color coded by spectral type. Control and RP subjects are in *A*–*C* and *D*–*F*, respectively. Control1 (*A*) and RP1 (*D*) cones were obtained from 6° retinal eccentricity (*SI Appendix*, Fig. S4 *A* and *D*); Control2 (*B*) and RP2 (*E*) cones were obtained from 4° retinal eccentricity (*SI Appendix*, Fig. S4 *B* and *E*); and Control3 (*C*) and RP3 (*F*) cones were obtained from 2° retinal eccentricity (*SI Appendix*, Fig. S4 *C* and *F*). Black lines are linear regression fits.

We examined the responses of 4,296 individual cones in six retinal patches (one from each control and RP subject) to test for a correlation between cone ΔOPL response and cone OS length. Results are shown in Fig. 10 for Control1 and RP1 at 6°, Control2 and RP2 at 4°, and Control3 and RP3 at 2°. Cones were classified and examined by spectral type. In the three controls (Fig. 10 *A*, *B*, and *C*), a significant positive effect was observed in six of nine conditions (three cone spectral types × three subjects), but the effect was weak in five of those six (*R*^2^ = 4.4, 6.6, 15, 1.1, and 2.3%) and strong in only one (*R*^2^ = 57%). The correlations were not significant in the remaining three conditions.

For the three RP subjects (Fig. 10 *D*, *E*, and *F*), we limited our analysis to cones that could be classified as M or L; weakly responding cones that located near the PC space origin could not be classified by spectral type and thus were labeled as undetermined in our plots. Of the 286 and 652 analyzed cones in RP1 and RP2, respectively, 263 (92%) and 567 (87%) were classified as M or L and were observed to have a significant positive relationship between cone ΔOPL response and OS length: for RP1, *R*^2^ = 60.9 (L) and 39.8% (M) and for RP2, *R*^2^ = 29.9 (L) and 36.5% (M).

For RP3, much fewer cones could be classified as M or L (38 out of 338 [11%] analyzed cones). M cones were observed to have a significant positive relationship between cone ΔOPL response and OS length (*R*^2^ = 35.6%), while L cones showed no significance. Considering the response of all the analyzed cones, the Fig. 10*F* plot for RP3 shows that while most cones responded weakly, their OS lengths varied more than sevenfold (4 to 31 μm). Interestingly, the strongest responding cones (those that could be classified) also had OS lengths that varied considerably (7 to 29 μm). Furthermore, the cones with the longest OSs (30 to 31 μm) had responses that were indistinguishable from the vast majority of other cones; in fact, we were unable to spectrally classify them.

In general, our results for the three controls and three RP subjects suggest that the relationship between cone ΔOPL response and OS length changes with cone cell health. We found little correlation between cone ΔOPL response and OS length in healthy cones (controls), an increased correlation in cones at patches afflicted with RP (RP1 and RP2), and little correlation in cones at the one severely diseased patch examined (RP3). At best, cone OS length appears to be a coarse predictor of cone ΔOPL response in our results, even among cones of the same spectral class.

Finally, it is interesting to note the dramatic impact RP has on the distribution of cone OS lengths as evident in the Fig. 10 histograms. For the controls, OS lengths are relatively uniform and Gaussian distributed. As expected for the RP subjects, OS lengths are generally shorter than for the age-matched controls. Unexpectedly, the distribution of OS lengths in the RP subjects is exceedingly wide, with cones as short as a few microns and others as long as those in controls. For RP1 and RP2, the distribution is bimodal, with peaks slightly shorter and much shorter than those in controls. For the most severely diseased patch examined (RP3), the distribution is unimodal but peaks at a short OS length of 7 μm. AO-OCT imaging exposes considerable variability in cone OS structure caused by this devastating disease that is missed by clinical OCT imaging.

### Attribution of Cone ΔOPL Response.

This study takes advantage of prior work that has established the ΔOPL response as an optical correlate of cone function (the ability to capture and transduce photons into visual signals) (22–26, 30–32). A growing consensus holds that the large increase (up to hundreds of nanometers) in the cone OPL after photostimulation is attributable to osmotic swelling, an increase in the cytoplasmic volume of the cone OS, and triggered by excess osmolytes produced by phototransduction. Direct evidence of this attribution remains limited (53); if it is correct, ΔOPL responses are a downstream effect of phototransduction and are thus likely to be affected by dysfunction at any stage of the phototransduction cascade occurring within the OS. This should make ΔOPL responses sensitive to a broad range of disease mechanisms that afflict photoreceptors, just as is hyperpolarization, the final functional response of the cone. This makes the ΔOPL response a potentially attractive biomarker for assessing the impact of disease severity on photoreceptor function.

On the other hand, we do not know how cones’ structural integrity (which could affect OS biomechanics) might be compromised by diseases such as RP. Our ΔOPL response would probably also capture abnormalities in the OS biomechanical response to osmotic stress due to the breakdown in structural integrity. A significant biomechanical effect could complicate our interpretation of the ΔOPL response and may require distinguishing changes in phototransduction from changes in OS biomechanics. Testing this idea would require comparing individual cone ΔOPL responses against the same cones’ hyperpolarization-induced electrical responses. While this was not possible in our study, the use of AO single-cell microperimetry might help by associating changes in the electrical responses of individual cone cells to changes in visual perception (11). Regardless, the results in our study show a clear trend: a decrease in ΔOPL response with increase in the degree of disease severity characterized using the TZ from clinical OCT and supported by clinical perimetry.

### Repeatability Error.

A cross-sectional study was performed to determine repeatability of our cone functional measurements. We divided the 12 videos acquired with each of the three (450, 528, and 637 nm) stimuli in RP1 at 6° into two sets (6 videos each). We mapped the OPL traces of the two sets using PC analysis, smoothed the 2D distribution of cone responses, and determined the peak density location of the smoothed distribution for the M- and L-cone clusters. The difference in the relative response strengths of the M and L clusters was less than 1 and 3%, respectively, indicating high repeatability (results in *SI Appendix*, Fig. S5). Of course, we would expect smaller errors if all 12 videos had been used.

### Therapeutic Applications and Future.

The recent explosion in gene replacement therapy, neuroprotective strategies, stem cell therapy, and optogenetics for RP and other inherited retinal degenerative diseases have placed increased demands on rapid outcomes and improved sensitivity to measure safety and efficacy. For these, our method may be useful in augmenting existing diagnostics for more precise assessment of the functionality of remaining retinal cells. For patient screening, this could be valuable in more precisely determining the extent to which existing photoreceptors can be rescued and thus the suitability of the different therapeutic options (7). Similarly, because RP is a slowly progressing disease, the ability of our method to measure dysfunctional changes in single cells may be particularly powerful in shortening the time to determine treatment efficacy. The inward progression of the TZ in RP is about 90 to 300 μm/y depending on mode of inheritance, retinal location, and study (54–56). OCT is one of the most sensitive clinical methods to measure progression, providing 95% test–retest reliability of 74 to 259 μm depending on the study (54–56); this yields a sensitivity to detect progression of about 6 to 36 mo. The ability of our method to track changes—now both structural and functional—in single cells could make it possible to detect progression on a much shorter time scale, from 0.4 to 1.1 mo. This sensitivity assumes the ability to detect the loss of a single-cone cell (of diameter 8 μm) and a typical rate of disease progression as reported in refs. 54–56.

Even when visual function is restored with gene therapy, the extent to which cone photoreceptors functionally and structurally recover is often unknown. Several early landmark clinical trials demonstrated the successful use of a gene augmentation technique to treat Leber congenital amaurosis, an inherited blinding disease that afflicts photoreceptors (57–59). This intervention provided at least short-term recovery of visual function (9), but surprisingly, this was not accompanied by cessation of structural degeneration of the cone photoreceptors (60). It has been hypothesized that this structure–function paradox is attributable to the survival of some healthy photoreceptors in the treated retina and the degeneration of others that may be in a preapoptic stress state. Our results in severely diseased patches support this hypothesis in that we find a small fraction of cones that still respond normally (see Fig. 9). The application of our method to treated subjects could directly test this hypothesis.

Furthermore, recent work in gene therapy increasingly requires measurements at the resolution limit of clinical imaging instruments in order to confirm posttreatment regeneration of retinal structures. For example, the first in-human gene therapy trial on X-linked RP caused by mutations in RPGR used clinical OCT to detect the apparent emergence of a new layer above the retinal pigment epithelium. This layer may represent regenerating photoreceptor OS, but it could not be identified as such because of insufficient resolution (61). Our AO-OCT method, with its much higher resolution and sensitivity, can provide considerably more detailed information about the suspected regrowth of photoreceptor OSs, including their functional state.

While our study with PS-AO-OCT was confined to changes in cone photoreceptors in RP subjects, our measurements have broader significance. PS-AO-OCT and variants of it are being extended to measure function in other retinal cells, including retinal pigment epithelium (62), rod photoreceptors (30), retinal ganglion cells (63), and neural connections in the inner plexiform layer (64). Continued development of the method raises the exciting possibility of tracking the evolution of cell dysfunction across the entire thickness of retinas afflicted by RP and other diseases.

## Materials and Methods

### Subjects.

We recruited three RP subjects whose diagnosis was based on the clinical appearance of the fundus, patient history, visual fields, and full-field electroretinogram results. The subjects had a best corrected visual acuity of 20/30 or better and a spherical equivalent refraction no greater than ±5 D to avoid signs of degenerative myopia. RP subjects were excluded if they had evidence of macular pathologies such as macular cysts or epiretinal membranes, a history of other ocular diseases (such as macular degeneration, diabetic retinopathy, or glaucoma), or clinical OCT images of poor quality. All subjects required clear ocular media, stable fixation, and a nonsyndromic inheritance (adRP, arRP, or X-linked RP). For imaging reasons, subjects were selected with a TZ between healthy and diseased retina that was located within 10° of the fovea center.

We also recruited three age-matched controls that were imaged at the same retinal locations as the RP subjects. These controls had no known pathology of the visual system and no previous history of clinically significant systemic disease. They had a best corrected visual acuity of at least 20/20 and a spherical equivalent refraction no greater than ±5 D to avoid possible complications due to degenerative myopia. As a second, larger control group, we pooled the cone responses from 14 healthy subjects that we previously reported, all at 3.7° retinal eccentricity (32). The 14 excluded 2 subjects that were also used in this study. Because some of the subjects had color vision anomalies (3 color normals, 3 deuteranopes, 2 protanopes, and 6 deuteranomalous trichromats), we pooled the S cone responses from all 14, the M cone responses from the 3 normals and 2 protanopes, and the L cone responses from the 3 normals, 2 deuteranopes, and 6 deuteranomalous trichromats. We used this group as a normative baseline to compare our three age-matched controls and three RP subjects.

### Procedures.

Measurements on the subjects included AO-OCT imaging, OCT imaging, genetic testing, color vision testing, eye length, and visual sensitivity. All procedures on the subjects adhered to the tenets of the Declaration of Helsinki and were approved by the Institutional Review Board of Indiana University. Written consent was obtained after the nature and possible risks of the study were explained. See *SI Appendix*, *SI Materials and Methods*.

### Experiment Design for AO-OCT Imaging.

The subject’s eye was cyclopleged and dilated with tropicamide 0.5% and aligned to the Indiana AO-OCT system (described in *SI Appendix*, *SI Materials and Methods*). AO-OCT volume videos (0.8° × 1°) were collected at four retinal eccentricities in all subjects: 2, 4, 6, and 8°, except at those that were too severely diseased to be used in this study. Depending on severity of the disease, these retinal eccentricities were located at the healthy central area (if present), at the border between the healthy area and TZ (if present), and within the TZ. Volumes were acquired at 10 Hz for 5 s; at 2.5 s, a 5-ms flash of visible light was delivered to the retina using one of the three light-emitting diode (LED) sources (peak energy at 450, 528, and 637 nm).

A total of 12 PS-AO-OCT volume videos were acquired at each location and each stimulus wavelength (450, 528, and 637 nm), one every 2 min over a 24-min period, in order to improve the signal-to-noise ratio by averaging. A total of 36 videos were acquired per location. Between videos, there was an empirically determined 90 s of dark adaption to reduce influence of photopigment bleaching that occurred during preceding videos.

### Postprocessing of AO-OCT Volumes.

Volumes were reconstructed, dewarped to correct nonlinearities in the fast-axis scan pattern, and registered to each other to correct eye motion artifacts. The latter achieved subcellular accuracy using our custom 3D registration algorithm (65) followed by semi-automatic identification of cones in the en face image. Registration entailed selecting one volume as a reference based on good image quality and minimum eye motion artifact. All volumes collected at the same retinal location were registered to this reference, regardless of stimulus protocol. Using a common reference allowed us to compare changes in the same cones under different stimulus conditions. Temporal changes in the OPL of each cone OS were extracted from the registered AO-OCT volumes using the complex form of the IS/OS and COST reflections (*SI Appendix*, *SI Materials and Methods*).

## Data Availability

All study data are included in the article and/or *SI Appendix*.
